# A Combinatory Therapy of Metformin and Dexamethasone Reduces the Foreign Body Reaction to Intraneural Electrodes

**DOI:** 10.3390/cells13242112

**Published:** 2024-12-20

**Authors:** Bruno Rodríguez-Meana, Jaume del Valle, Xavier Navarro

**Affiliations:** 1 Institute of Neurosciences, Department of Cell Biology, Physiology and Immunology, Universitat Autònoma de Barcelona, 08193 Bellaterra, Spain; 2 Secció de Fisiologia, Departament de Bioquímica i Fisiologia, Universitat de Barcelona, 08028 Barcelona, Spain; 3 Centro de Investigación Biomédica en Red en Enfermedades Neurodegenerativas (CIBERNED), 28029 Madrid, Spain

**Keywords:** foreign body reaction, neuroprostheses, metformin, dexamethasone, macrophages, fibroblasts

## Abstract

Neural electrodes used for bidirectional communication between the nervous system and external devices like prosthetic limbs have advanced in neuroprosthetic applications. However, their effectiveness is hindered by the foreign body reaction, a natural immune response causing inflammation and fibrosis around the implanted device. This process involves protein adsorption, immune cell recruitment, cytokine release, and fibroblast activation, leading to a fibrous capsule formation and a decrease in electrode functionality. Anti-inflammatory and antifibrotic strategies have the potential to diminish the impact of the foreign body response. In this work, we have evaluated long-term metformin administration and short-term dexamethasone administration as a combined therapy to modulate the foreign body reaction induced by a polyimide intraneural implant in the sciatic nerve of rats. After a 12-week implant, the foreign body reaction was significantly reduced only in the group administered both drugs.

## 1. Introduction

Neuroprostheses have increasing interest in the field of neuroscience, as they provide potential to restore the lost neural functions after a nerve injury or loss of a limb. Neural electrodes are used to create a bidirectional communication interface between the peripheral nervous system and an external device, such as a prosthetic limb [1]. However, the use of these implants is limited by the foreign body reaction (FBR), leading to inflammation and fibrosis around the implanted device, that increases tissue resistance and reduces the functionality of the electrode.

The FBR is a complex biological process that occurs when a foreign object is introduced into the body, as a natural response of the immune system to protect the body from potential harm caused by the foreign element. This reaction involves a series of events, beginning with the adsorption of proteins onto the surface of the implant and the recruitment and activation of immune cells. Immune cells then release cytokines and chemokines that promote inflammation and recruit fibroblasts, which deposit collagen around the implant site. Over time, this process concludes with the formation of a dense fibrous capsule around the implant, leading to increased impedance, increased currents needed to stimulate the axons, signal recording attenuation, and in some cases, implant failure [2,3,4,5,6]. Tissue–electrode mismatch and the micro-motion of the intraneural device also contribute to the formation of the fibrotic capsule. Despite that the materials may be well tolerated, the relative mechanical motion appears to increase the thickness of connective tissue around implanted cuff electrodes [7]. Chronic studies with intraneural TIME electrodes have proved that electrical stimulation does not contribute to enhance the FBR, whereas insertion trauma and chemical/mechanical mismatch may play a major role in the process [8]. In this sense, it is worth noting that motion and tethering forces affect electrode implants more in the peripheral nerves than in the brain.

Several strategies have been proposed to reduce the FBR and improve the biocompatibility and long-lasting functionality of neural implants. These strategies include using more flexible materials as the electrode substrate, introducing surface modifications of the implant material, coatings with biomimetic hydrogels, and the local release or most often systemic administration of anti-inflammatory or antifibrotic drugs, among others [3,6,9]. Dexamethasone is a corticosteroid that has been used to reduce the FBR to neural implants. Dexamethasone can be administered systemically or locally, such as using coatings or hydrogels, to reduce inflammation and fibrosis around the implant. The systemic administration of dexamethasone has been proven to reduce the capsule around intraneural devices [10] and significantly improve chronic functionality [4]. However, there are concerns about the side effects of long-term use of systemic corticosteroids, including osteoporosis and metabolic and cardiovascular disease [11]. On the other hand, metformin is a widely used drug for type II diabetes that has been shown to prevent fibrosis in different tissues. This antifibrotic effect is attributed to adenosine monophosphate-activated protein kinase (AMPK) activation, which interferes with transforming growth factor-β1 (TGF–β1) signaling, a key pathway involved in fibrosis [12]. While the effects of metformin on fibrosis in peripheral nerves have not been previously studied, the results seen in other tissues suggest that it may be a promising candidate for reducing the formation of the fibrotic capsule around intraneural implants. To test this hypothesis, we used intrafascicular polyimide devices longitudinally implanted in the sciatic nerve of rats, and treated them systemically with metformin and with dexamethasone for comparison.

## 2. Materials and Methods

### 2.1. Surgical Procedure

Surgeries were conducted on female Sprague–Dawley (SD) rats weighing 300–350 g, using ketamine/xylazine (90/10 mg/kg i.p.) as anesthesia. The sciatic nerve was exposed at the mid-thigh level and freed from surrounding tissues. A thin-film device of polyimide (PI) was longitudinally implanted in the tibial branch of the sciatic nerve with a straight needle attached to a 10–0 loop thread (STC–6, Ethicon, San Lorenzo, PR, USA), as described for the longitudinal intrafascicular electrodes (LIFEs) [13,14]. The insertion was performed under a dissection microscope to verify correct placement of the device. After surgery, all animals were housed under standard conditions and periodically subjected to functional and electrophysiological evaluations. The incision wounds healed without inflammatory signs and no postoperative complications were observed in any of the rats with the intraneural implant.

At predesigned intervals of 2, 8, and 12 weeks, subgroups of 5–7 animals were euthanized, and the sciatic nerve was harvested for histological studies. Animal experiments were performed following protocols approved by the Ethical Committee of the Universitat Autònoma de Barcelona in accordance with the European Communities Council Directive 2010/63/EU.

### 2.2. Drug Administration

Treatments for modulating the FBR against intraneural PI devices started 2 days prior to the surgery to ensure adequate systemic levels. We assayed three therapies, metformin to reduce fibroblast activation, dexamethasone to reduce macrophage activation, and the combined administration of both. The distribution of animals in groups, doses, and administration pathways are summarized in Table 1. Metformin (MET) (Qualigen, Neuraxpharm, Barcelona, Spain) was administered in the drinking water (p.o.) (125 mg/kg/day) up to 12 weeks. The dosage was selected according to human use and considering the amount of water that SD rats drink per day. Dexamethasone (DEXA) (Kern Pharma, Terrassa, Spain) was administered subcutaneously (s.c.) once a day for 2 weeks at 0.2 mg/kg/day according to a previous report [10]. One group received metformin alone. A second group was treated with both metformin and dexamethasone (DEXA + MET). A third group was given only dexamethasone. This last group was conceived in a previous work in our laboratory [10] but histological quantifications were performed again. The control (CTL) group did not receive any treatment.

### 2.3. Electrophysiological and Functional Evaluation

The functional properties of the implanted nerves were assessed through nerve conduction studies, algesimetry, and locomotion tests performed at 2, 8, and 12 weeks post-implantation. The contralateral side served as control. For nerve conduction tests, the sciatic nerve was stimulated proximally with single electrical pulses and the compound muscle action potential (CMAP) was recorded from gastrocnemius (GM) and plantar interossei (PL) muscles as previously described [14,15]. The nociceptive threshold to mechanical stimuli was assessed using an electronic Von Frey algesimeter (Bioseb, Chaville, France) following a published protocol [16]. The rats were positioned on a wire mesh platform within plastic chambers, and a metal tip was applied to the sole of the hindpaw until a withdrawal response was elicited. The applied force at the time of withdrawal was noted. To evaluate locomotor function, the walking track test was conducted. The plantar surface of the hindpaws was painted with blue ink, and the rats were allowed to walk through a corridor lined with white paper, leaving imprints for the analysis. The print length and the distance between the 1st and 5th toes and between the 2nd and 4th toes were measured to calculate the sciatic functional index (SFI) [17].

### 2.4. Histological Evaluation

At 2, 8, and 12 weeks, subgroups of animals were injected with an overdose of pentobarbital and perfused transcardially with 4% paraformaldehyde (PFA) in a phosphate buffer (PB). The sciatic nerve, along with the implanted device, was carefully extracted, and postfixed in 4% PFA for one hour, and then a segment was immersed in 30% sucrose in PB for cryoprotection.

To evaluate macrophage infiltration and capsule thickness around the implant, immunohistochemical labeling was performed. Nerve segments containing the PI device were cryosectioned into 15 µm thick slices using a cryostat (Leica CM190; Deer Park, IL, USA). After thawing and blocking with normal donkey serum, the sections were incubated overnight at 4 °C with the following primary antibodies: rabbit anti-Iba1 (1:500; Wako, Osaka, Japan) to label macrophages, RT97 (1:200; Developmental Studies Hybridoma Bank, Iowa City, IA, USA) for axons, and cluster of differentiation 90 (CD90) (1:150; BD Pharmingen, Franklin Lakes, NJ, USA) to identify fibroblasts. The sections were washed with PBS containing 0.1% Tween 20 and then incubated for 1 h at room temperature with secondary antibodies AlexaFluor 488 donkey anti-mouse and AlexaFluor 555 donkey anti-rabbit (Invitrogen, Thermo Fisher, Waltham, MA, USA). The slides were mounted with Mowiol containing DAPI (Sigma, Merck KGaA, Darmstadt, Germany) for nuclear staining.

The number of Iba1-positive macrophages in the tibial nerve was semiautomatically quantified using a custom-made macro for Image J software (v1.8, National Institutes of Health). The workflow begins with image preprocessing, including brightness and contrast adjustment for consistency across image sets. Thresholding is then applied automatically to segment Iba1+ regions, followed by the creation of a binary mask and application of the watershed function to separate overlapping cells. The macro integrates pre-saved regions of interest (ROIs) to focus the analysis exclusively on the tibial fascicle, excluding the area corresponding to the implanted device. Using the “Analyze Particles” function, cells are identified and counted with constraints on size (≥90 pixels) and circularity (0.4–1.0). The mean capsule thickness was analyzed by dividing the area of the capsule around the implant by the length of the implant in the transversal section. The area was quantified as the non-labeled space between the implant and the first axons labeled with 200 kDa clone RT97. The delineated ROI excluded any tissue-empty areas due to the sectioning process. As each PI implant has two arms, the capsule thickness of an implant was calculated as the mean of both arms. Images were taken with an epifluorescence microscope (Eclipse Ni, Nikon, Amstelveen, The Netherlands) and a digital camera (DS–Ri2, Nikon).

Masson trichome staining was also performed in other nerve cross-sections according to standard protocols to label the deposited collagen.

Another segment of the implanted nerves was processed for a light microscopy analysis by embedding in epon resin. These samples were fixed in a solution containing 3% glutaraldehyde and 3% paraformaldehyde, then postfixed in 2% OsO_4_ for 2 h. Following dehydration through a graded ethanol series, the samples were embedded in epon resin. Thin sections (0.5 μm) were cut with an ultramicrotome, stained with toluidine blue, and viewed under light microscopy. Images were captured and transformed to greyscale. For each animal, a section was selected, and the capsule thickness was measured as the distance from the implant to the nearest myelinated axon using ImageJ software.

### 2.5. Data Analysis

The results are shown as the mean ± standard error of the mean (SEM). The normality of the data was tested with the Shapiro–Wilk test. Statistical comparisons between groups were made by two-way ANOVA followed by Tukey’s multiple comparison test. Differences were considered significant when *p* < 0.05. In the correlation graphs, the linear regression is represented as a solid line, and the shaded area represents the 95% CIL. The Pearson correlation coefficient was used to evaluate the strength of the correlation. GraphPad Prism 8 software was used for statistical analyses.

## 3. Results

### 3.1. Functional Evaluation

**Figure 1:**
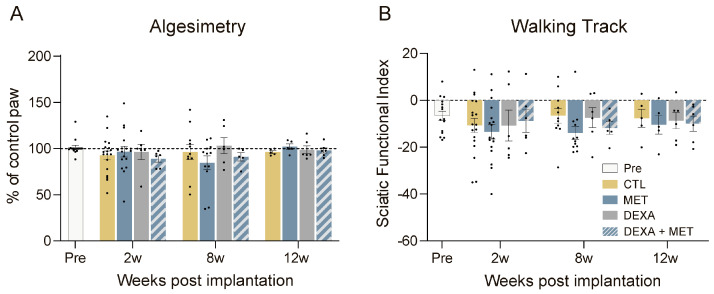
Results of the functional tests in rats with a PI device implanted in the tibial nerve. (**A**) Algesimetry test results expressed as percentages of force thresholds for withdrawal (vs. contralateral control paw) of animals before the implantation and after the implantation and treatments for 12 weeks. (**B**) The plot of the SFI obtained in the walking track test. No significant differences were found.

The algesimetry tests yielded a pain withdrawal threshold close to the preoperative test in the implanted and contralateral hindlimbs in all the groups along the follow-up (Figure 1A), with no evidence of hyperalgesia that could suggest nerve injury. Similarly, the walking track analysis (Figure 1B) showed no differences between the three groups at any time point. The SFI values remained close to zero, which is considered normal, throughout the study. In conclusion, there was no evidence of alterations in sensory and motor functions conveyed by the sciatic nerve after the PI device was implanted, and the administered drugs did not affect this outcome.

**Figure 2:**
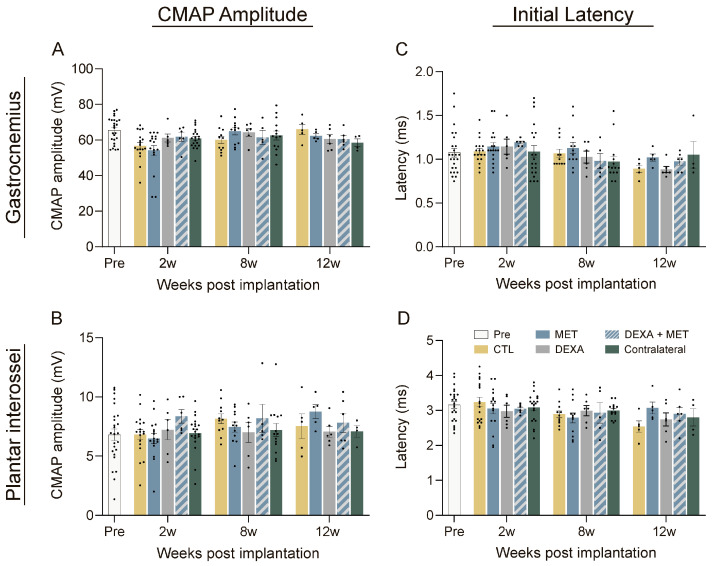
Results of the functional tests in rats with a PI intraneural device implanted in the tibial nerve. Motor nerve conduction parameters of animals before implantation (Pre) and after the implantation of PI devices for 12 weeks and drug administration. (**A**,**B**) CMAP amplitudes of GM (**A**) and PL (**B**) muscles. (**C**,**D**) CMAP onset latencies of GM (**C**) and PL (**D**) muscles. No significant differences were found in electrophysiological test results.

No significant changes in the electrophysiological results of the three groups of rats (CTL, MET, and DEXA + MET) were detected in the nerve conduction tests after the implantation of the device. The amplitude of the CMAPs (Figure 2A,B) of the implanted animals did not show significant variations in comparison with the contralateral paw and with the untreated CTL group at any time point, thus indicating that there was no functional damage to the nerve. In some animals, there was a slight decrease in CMAP amplitude at 2 weeks, which can be attributed to the surgical procedure alone and not to the implant since it recovered a few weeks later, as reported in other studies using similar longitudinal implants [10,14]. The latency of the CMAPs did not show significant differences between groups during the follow-up (Figure 2C,D), indicating that there was not demyelination or focal compression affecting the impulse conduction velocity.

### 3.2. Inflammatory Response

**Figure 3:**
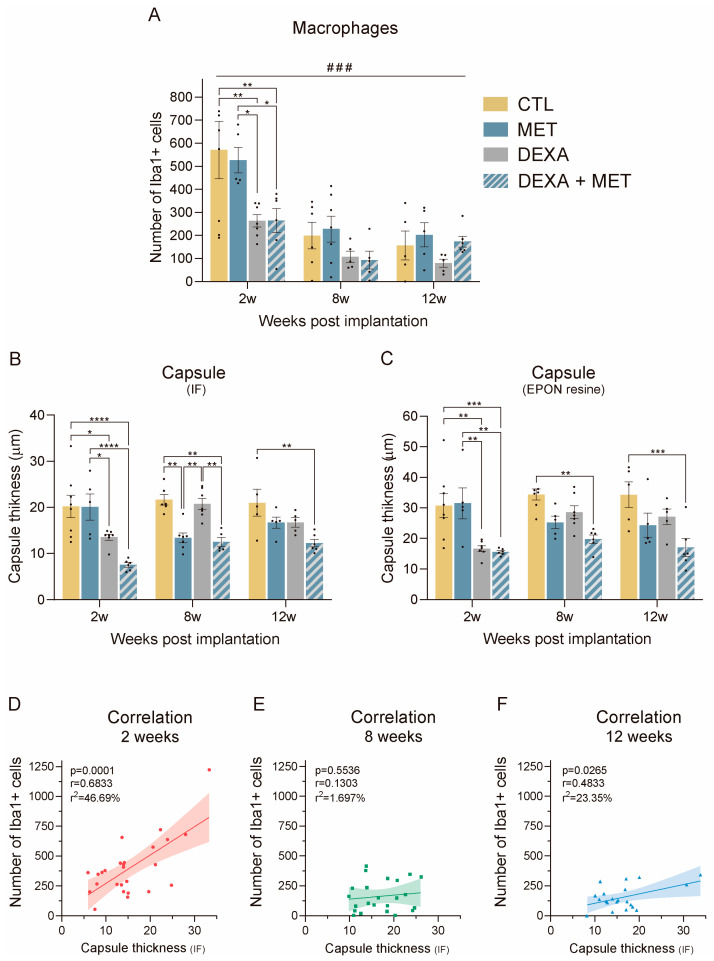
The effect of drug administration on the FBR to intraneural implants. (**A**) The number of inflammatory Iba1+ cells in the tibial nerve of animals implanted with PI devices and administered metformin, dexamethasone, or both. (**B**,**C**) Tissue capsule thickness around the devices in the tibial nerve of animals implanted with PI receiving the different treatments. Measurements were made using immunofluorescence sections (**B**) and thin sections of epon-embedded nerves (**C**). (**D**–**F**) Correlation between the number of Iba1+ cells and capsule thickness (IF) at 2, 8, and 12 weeks after implantation. The solid lines represent the linear regression, while the shaded area represents the 95% CIL. * *p* < 0.05, ** *p* < 0.01, *** *p* < 0.001, **** *p* < 0.0001, and ### *p* < 0.01 time variable, two-way ANOVA followed by Tukey’s multiple comparison test.

**Figure 4:**
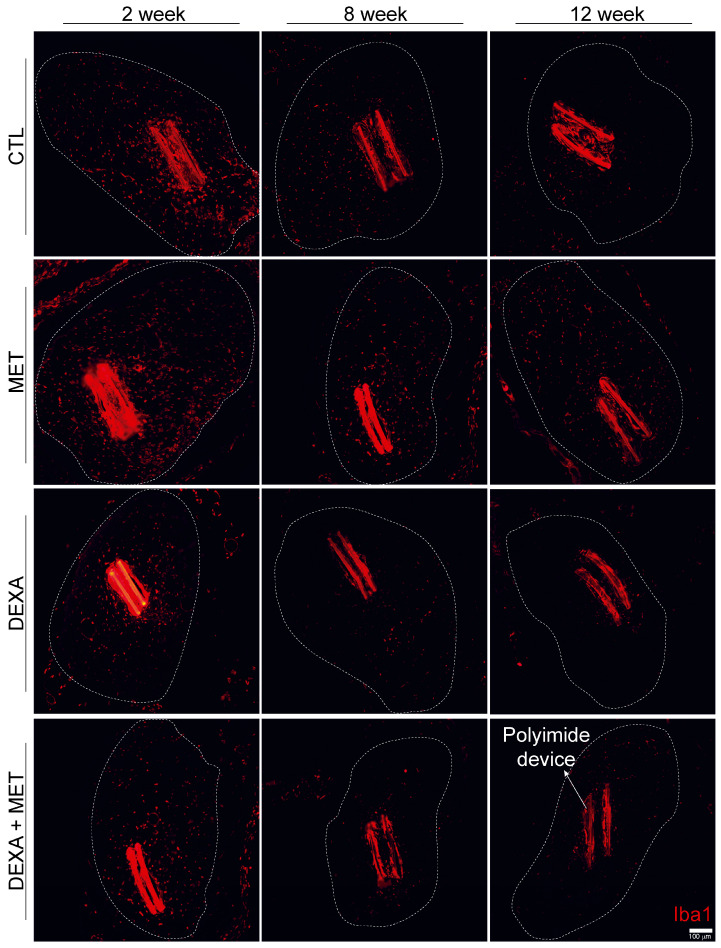
Representative images of inflammatory cells (red, Iba 1+ cell) infiltrating the tibial nerve after 2, 8, and 12 weeks of the PI intraneural device implantation in the different groups studied. Note the intense fluorescence emitted by the PI. The area limited by the dotted line corresponds to the tibial fascicle of the sciatic nerve that was used to analyze the number of labeled cells. Scale bar: 100 μm.

During the initial phase of the FBR, macrophages are the predominant reactive cells. In the case of intraneural longitudinal implants, a peak of infiltrating macrophages was observed around 2 weeks after implantation [14]. We also found a high number of macrophages in the tibial nerve fascicle at 2 weeks of the PI device implantation (Figure 3A and Figure 4). The groups administered dexamethasone (alone or combined with metformin) exhibited significantly fewer macrophages at 2 weeks, but not the group receiving only metformin. As the FBR resolves, the number of macrophages decreases around the implant and in the nerve regardless of treatment. Notably, at 8 weeks, the number of macrophages in the groups administered dexamethasone for 2 weeks remained lower, although not significantly, compared to the groups not receiving dexamethasone. Therefore, while metformin did not affect the number of macrophages present in the tibial nerve, 2-week daily treatment of dexamethasone significantly reduced the infiltration of hematogenous macrophages, even at late times of 8 and 12 weeks. Figure 4 shows representative immunofluorescence images of labeled macrophages for all treatments and studied time points.

### 3.3. Capsule Formation

**Figure 5:**
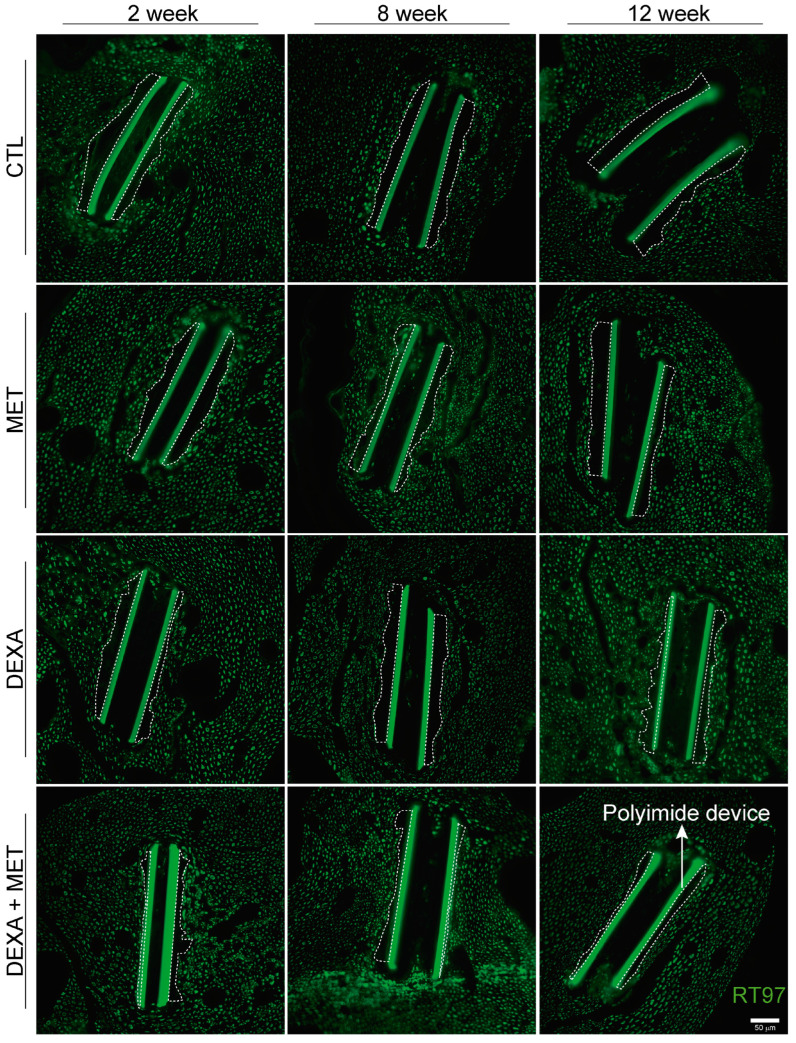
Representative images of nerve cross-sections around the PI intraneural implant after 2, 8, and 12 weeks of the implantation in the different groups studied. Nerve fibers are labeled with antibody RT97. Note the intense fluorescence emitted by the PI. The measured capsule surrounding the PI device is the area delimited by the dotted line, which separates the PI from the nerve fibers, excluding tissue-empty regions. Scale bar: 50 μm.

**Figure 6:**
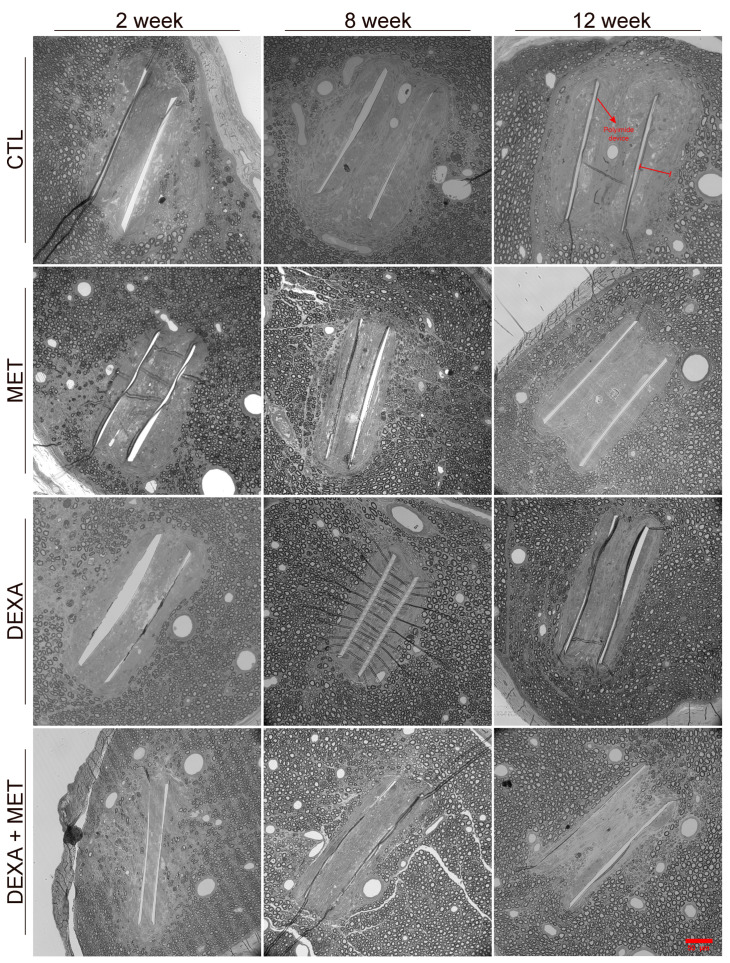
Representative images of cross-sections of the nerves embedded in epon resin and stained with toluidine blue, corresponding to samples taken at 2, 8, and 12 weeks for the different study groups. The images show the PI implants (pointed to by a red arrow in the top-right panel) within the nerve, surrounded by the capsule and axons. The thickness of the capsule from the implant to the first axons is marked with a red bar in the top-right panel. Images were acquired and transformed to greyscale. Scale bar: 50 μm for all the panels.

**Figure 7:**
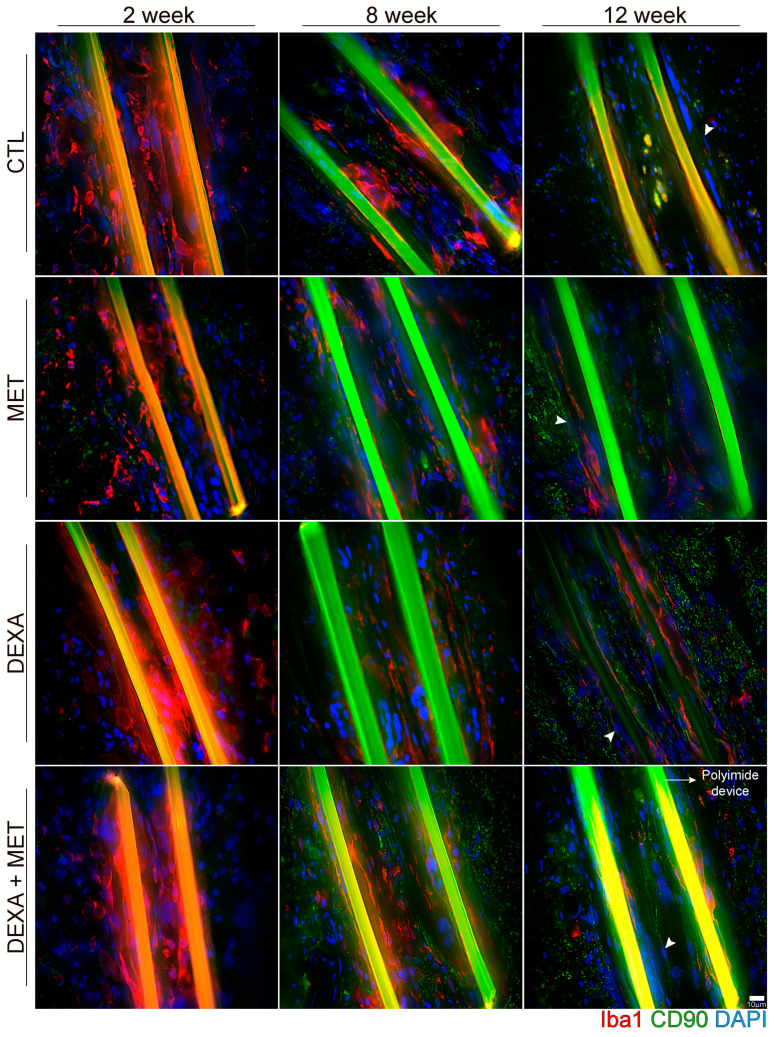
Representative images of the capsule composition around the PI intraneural implant. Immunohistochemical labeling for macrophages (red, Iba 1+), fibroblasts (green, CD90, arrowheads), and nuclei (blue, DAPI) of tibial nerves of animals of the different groups implanted with a PI device after 2, 8, and 12 weeks. Scale bar: 10 μm. Images with the individual channels are presented as Supplementary Materials Figures S1–S3.

**Figure 8:**
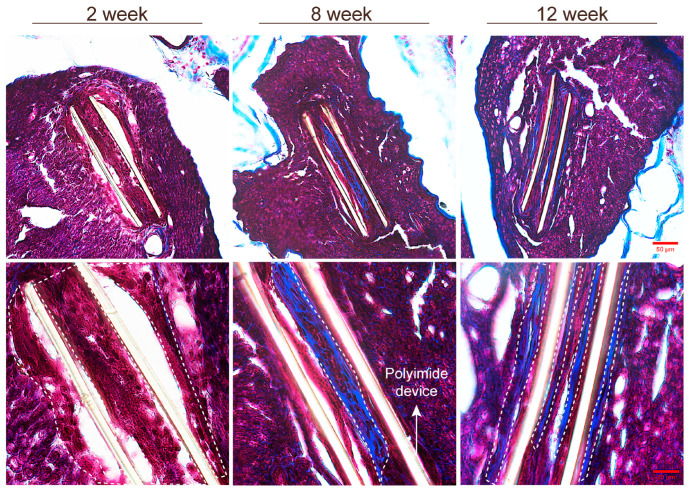
Representative images of nerve sections stained with Masson’s trichrome stain, showing the deposition of collagen in the capsule around the PI intraneural implant. At 2 weeks, the pink-stained area, outlined by the dotted line, corresponds to macrophages around the implant. At 8 and 12 weeks, the pink areas around the devices decreased, while the blue-stained areas (dotted line), composed of collagen fibers, were more preeminent surrounding the implant. Scale bar: 50 and 20 μm.

In the early phases of FBR, the capsule surrounding the device is made up mainly of macrophages that adhere to the implant trying to engulf it, whereas fibroblasts appear later, covering the implanted device with a fibrotic capsule [14]. Consequently, a positive correlation (r = 0.683) was found at 2 weeks (Figure 3D) between the capsule thickness (IF) and the number of macrophages, which declined at 8 and 12 weeks (Figure 3E,F). Thus, the groups administered dexamethasone had reduced thickness of the capsule at 2 weeks (Figure 3B,C), because of the decreased number of infiltrating macrophages (Figure 3A). On the contrary, the group receiving only metformin did not show such a reduction in capsule thickness at this time point. At the 8- and 12-week time points, as the macrophages leave the implant surface, the capsule becomes less cellular and more fibrous and compact (Figure 5 and Figure 6) due to the increased presence of fibroblasts (Figure 7), and the deposition of collagen fibers appears in the capsule (Figure 8). At 8 weeks, the capsule thickness was significantly lower in the two metformin-administered groups, but not in the dexamethasone-alone group, compared to the control group. In contrast, the quantification performed in epon resin showed that at 8 weeks, only the group treated with both dexamethasone and metformin had a significantly thinner capsule compared to the control group. At 12 weeks, only the group with both dexamethasone and metformin had a significantly thinner capsule than the control group.

Although the absolute values were slightly different on histological sections taken under immunofluorescence and under light microscopy due to differences in processing, the comparative results along time and among the groups were similar for the two methods of quantification.

## 4. Discussion

The results of this study corroborate that the systemic administration of dexamethasone decreased macrophage infiltration around the PI intraneural implant, as it has been described previously [10,18]. Therefore, the thickness of the capsule was reduced during the initial phase. The action of metformin targets fibroblast and myofibroblast actions, which become noticeable at the late phase of the FBR. Consequently, the capsule thickness decreased from 8 weeks with metformin administration while dexamethasone alone lost its initial preventing effect. Conversely, the reduction in capsule thickness at 12 weeks was mostly evident with the combined treatment of dexamethasone and metformin, suggesting a summatory effect of both drugs.

The reduction in functionality associated with the FBR is a relevant problem for the chronic application of neural interfaces [2,4,6]. Strategies aimed at mitigating the FBR have focused on two main approaches: the first involves the use of biocompatible materials or the optimization of physicochemical properties of materials to enhance their biocompatibility. This can include altering the shape, size, rigidity, and wettability of the materials. The second approach involves the administration of drugs, either systemically or locally, to modulate the FBR [19]. Local administration systems have significantly advanced in recent years, allowing control of drug release by certain stimuli (electrical, pH, light irradiation, etc.) [20,21], or the sequential delivery of multiple drugs [22]. Local delivery systems have been used in implants in the CNS [23,24], but less in the PNS using cuff or regenerative electrodes [19,25,26,27]. In contrast, drug coatings for intraneural PNS electrodes have not been explored in vivo [28,29]. The increase in size and thickness of the electrode substrate resulting from the addition of coatings or microfluidic systems should be taken into account, because of the volume constraints and the increase in endoneurial pressure of peripheral nerves.

We selected systemic administration since it allows the combination of various FBR-modulating drugs at different stages following implantation, according to the expected time window of effectiveness. We found that metformin reduces the thickness of the capsule deposited around the implant at the midterm. Furthermore, a combination treatment with dexamethasone and metformin reduces the inflammatory infiltration during the first phase and maintains a thinner capsule over the long term.

Dexamethasone has been demonstrated to reduce the FBR against different neural implants [4,10,18,19,30]. It acts through the glucocorticoid receptor, decreasing the expression of inflammatory mediators and reducing the recruitment and activation of macrophages [31,32]. Therefore, its maximum effect is expected to occur during the initial phase of the FBR when macrophages are the predominant cells, and the environment is pro-inflammatory. However, a long administration of dexamethasone causes several metabolic disturbances [11]. Therefore, the selected doses for our study are within the low range administered to humans [33], which are associated with fewer side effects. Additionally, efforts are made to limit the duration of administration. In this regard, an administration period of 2–4 weeks is sufficient to achieve a reduction in the inflammatory reaction and the capsule around the implant; however, a loss of effect is expected compared to long-term administration [10], as also observed in the present study.

Metformin, commonly prescribed for lowering blood glucose in patients with type II diabetes, has gained attention in recent years for its potential as an antifibrotic agent in several in vitro and in vivo models [12,34,35]. The antifibrotic effect of metformin is attributed to its impact on the TGF–β signaling pathway, and on reducing oxidative stress [36,37]. Of particular importance is the disruption of the TGF–β1 pathway resulting from the activation of AMPK, leading to the downregulation of fibrosis-associated genes, which reduce the production of collagen and fibronectin [38,39]. Additionally, AMPK activation and the decrease in reactive oxygen species (ROS) production by metformin reduce the migration and activation of fibroblasts and deactivate activated myofibroblasts [40,41].

This is the first work investigating the effect of metformin on the FBR elicited by an intraneural device. The daily administration of metformin for 12 weeks did not show any noticeable effects on health status of the rats (weight and behavior), indicating that metformin can be given in chronic systemic regimes. The antifibrotic effect exerted by metformin was noticeable at 8 weeks, but at 12 weeks, the capsule thickness enlarged and was not different to that of the control group. Most studies with metformin showing an antifibrotic effect in vivo involved sub-chronic administrations lasting 1 week [42,43], 2–3 weeks [12,35,40,44,45,46,47], 4 weeks [48,49,50,51], and 8 weeks [52,53]. It is important to mention that in our model, we did not observe a reduction in fibrosis at 2 weeks because fibroblasts and collagen production are not appreciable surrounding the implant until 8 weeks [15]. Moreover, the role of myofibroblasts in the fibrosis associated with peripheral nerve injuries appears to be focused on collagen contraction rather than production. Myofibroblasts are the main producers of collagen in other tissues, but their presence in the nerve is very scarce [54]. Therefore, metformin may not be exerting its full antifibrotic potential in the peripheral nerve, or higher doses may be needed along the duration of the implant. Regarding the anti-inflammatory action of metformin, we did not find a reduction in the number of infiltrated macrophages in the nerve. However, other studies have demonstrated anti-inflammatory effects, including the reduction in macrophage activation and recruitment, promotion of their differentiation into M2-like macrophages, or the reduction in cytokine production [43,53,55,56,57]. Recently, a study showed that the daily intraperitoneal administration of metformin, together with two other compounds acting as a metabolic inhibitor cocktail, significantly reduced the connective capsule thickness surrounding subcutaneously implanted cellulose disks at 2 weeks and more so at 4 weeks of the implant [58]. Interestingly, they found that the cocktail induced a general state of quiescence in the capsule-associated transcriptome, with the most relevant changes being the upregulation of pathways of TGF-β signaling and biosynthesis of unsaturated fatty acids, and gene changes that contribute to wound healing and reduced scar formation. Indeed, similar to macrophages, dermal profibrotic fibroblasts are metabolically dependent on glycolysis, and antifibrotic cells are dependent on fatty acid oxidation, suggesting a positive action of metformin treatment [59].

We have also assessed a combined treatment to modulate the two different stages of the FBR. During the early phase of the FBR, the pro-inflammatory environment primarily generated by macrophages initiates a feedback process in which pro-inflammatory mediators recruit more macrophages that surround the implant site. Since the implant material cannot be eliminated, macrophages transition from a pro-inflammatory phenotype to an anti-inflammatory one and lay the groundwork for the deposition of foreign body giant cells and the predominant action of fibroblasts during the late phase of the FBR [14]. These cells secrete extracellular matrix (ECM) components such as fibronectin and collagen, leading to the formation of a capsule that isolates the implant from the tissue [6]. Dexamethasone reduces the number and activation of macrophages, promoting an anti-inflammatory environment. Simultaneously, metformin reduces the action of fibroblasts, resulting in a smaller capsule around the implant. As hypothesized, this combined treatment was the most effective compared to separate metformin and dexamethasone administration.

The benefits of the combined treatment reported in this study must be proven in further studies, assessing if this reduction in the FBR is reflected in reduction in tissue impedance and improvement of stimulation and recording properties of the electrodes [4]. However, to reach this evaluation, the model of a passive device implantation, mimicking physically a “real” electrode as in our model, is very useful for initial screening and for finding the adequate dosage [10].

Whereas the phenotyping of macrophages differentiating the pro- and the anti-inflammatory states is possible with cell labeling, the distinction of the activated state of fibroblasts is more complicated; indeed, the fibroblasts that penetrate the nerve and surround the intraneural device are not marked with common antibodies against vimentin, smooth muscle actin, and others [14]. Our combined treatment showed synergic effects of the two compounds used, and paves the way to search for other more complex combinations, and also to develop focal administration paths to reduce potential secondary complications of the drugs given systemically [58].

## 5. Conclusions

Metformin is a promising antifibrotic treatment that can be safely administered chronically. The fibrotic capsule around the intraneural implant decreased over the medium term, although the effect declined over the long term. A combined therapy of dexamethasone and metformin appears to be an effective strategy to modulate the complex process of the FBR in the peripheral nerves.

## Figures and Tables

**Table 1:** Groups and treatments given in this study.

Drug Group	Treatment Duration	Implant Duration	Dose	Administration	n
CTL	–	2 w	–	–	7
		8 w			6
		12 w			5
MET	2 w	2 w	125 mg/kg/day	p.o.	5
	8 w	8 w			7
	12 w	12 w			5
DEXA	2 w	2 w	0.2 mg/kg/s.i.d.	s.c.	7
		8 w			7
		12 w			6
DEXA + MET	2 w (D) + 2 w (M)	2 w	0.2 mg/kg/s.i.d. (D) 125 mg/kg/day (M)	s.c. (D) p.o. (M)	6
	2 w (D) + 8 w (M)	8 w			5
	2 w (D) + 12 w (M)	12 w			5

p.o.: oral; s.c.: subcutaneous; s.i.d.: once a day; DEXA, D: dexamethasone; MET, M: metformin.

## Data Availability

Data will be made available from the authors under reasonable request.

## Supplementary Materials

The following supporting information can be downloaded at: https://www.mdpi.com/article/10.3390/cells13242112/s1, Figure S1: Representative images of the capsule composition around the PI intraneural implant. Immunohistochemical labeling for nuclei (blue, DAPI) of tibial nerves of animals of the different groups implanted with a PI device after 2, 8 and 12 weeks. Scale bar: 10 μm; Figure S2: Representative images of the capsule composition around the PI intraneural implant. Immunohistochemical labeling for fibroblasts (green, CD90) of tibial nerves of animals of the different groups implanted with a PI device after 2, 8 and 12 weeks. Scale bar: 10 μm; Figure S3: Representative images of the capsule composition around the PI intraneural implant. Immunohistochemical labeling for macrophages (red, Iba 1) of tibial nerves of animals of the different groups implanted with a PI device after 2, 8 and 12 weeks. Scale bar: 10 μm.
