# Supplementary material for: A Combinatory Therapy of Metformin and Dexamethasone Reduces the Foreign Body Reaction to Intraneural Electrodes
Source: Cells. 2024 Dec 20;13(24):2112. doi: 10.3390/cells13242112 (PMC11726768; doi:10.3390/cells13242112)
Supplement: Supplementary file 1 [file cells-13-02112-s001.zip › Caption sup figures.pdf]

Supplementary Figure S1. Representative images of the capsule composition around the PI intraneural implant. Immunohistochemical labeling for nuclei (blue, DAPI) of tibial nerves of animals of the different groups implanted with a PI device after 2, 8 and 12 weeks. Scale bar: 10 µm.

Supplementary Figure S2. Representative images of the capsule composition around the PI intraneural implant. Immunohistochemical labeling for fibroblasts (green, CD90) of tibial nerves of animals of the different groups implanted with a PI device after 2, 8 and 12 weeks. Scale bar: 10 µm.

Supplementary Figure S3. Representative images of the capsule composition around the PI intraneural implant. Immunohistochemical labeling for macrophages (red, Iba 1) of tibial nerves of animals of the different groups implanted with a PI device after 2, 8 and 12 weeks. Scale bar: 10 µm.
