# Supplementary figures and images for: A Combinatory Therapy of Metformin and Dexamethasone Reduces the Foreign Body Reaction to Intraneural Electrodes
Source: Cells. 2024 Dec 20;13(24):2112. doi: 10.3390/cells13242112 (PMC11726768; doi:10.3390/cells13242112)

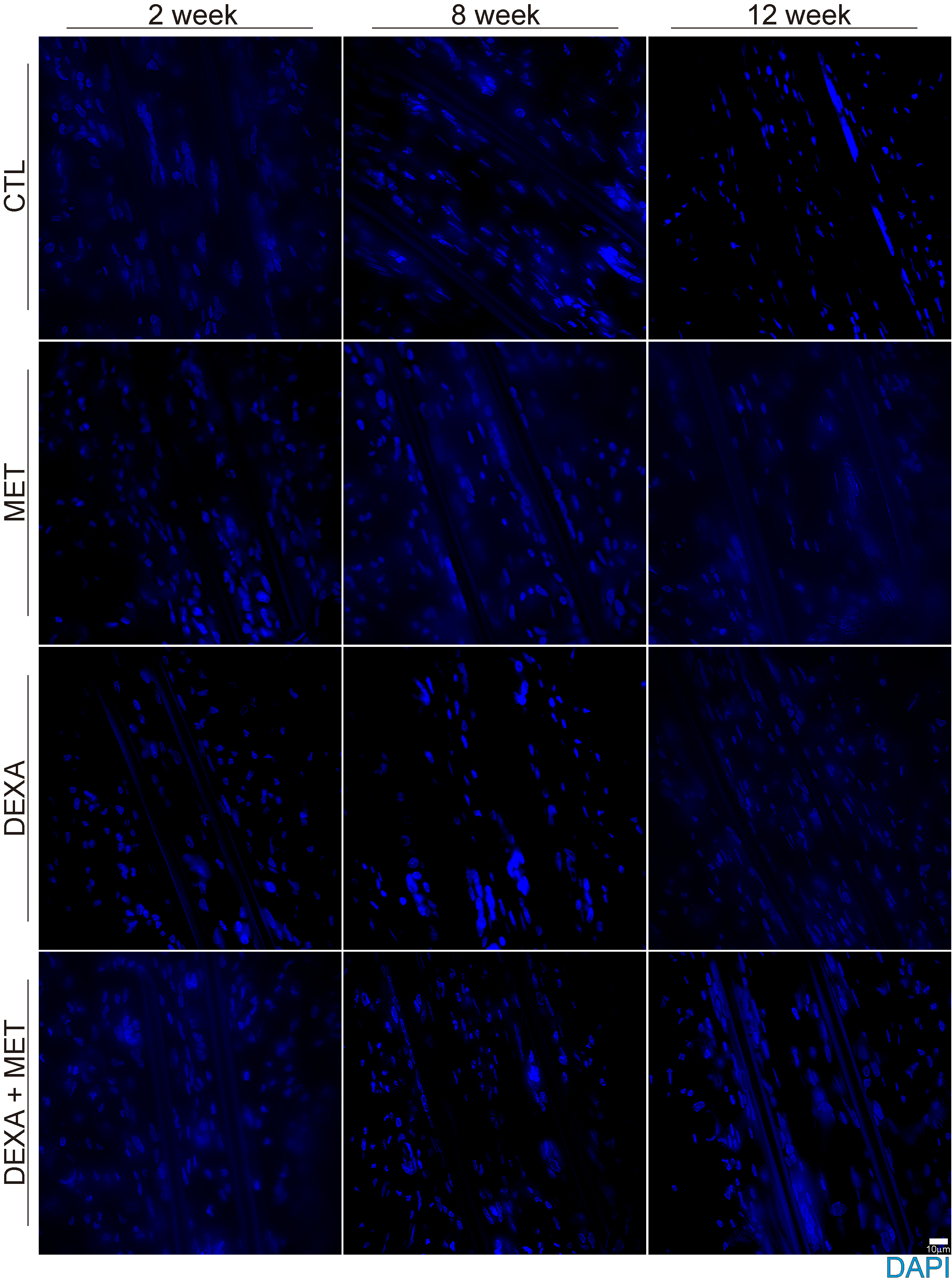

Supplement: Supplementary file 1 [file cells-13-02112-s001.zip › Sup Figure S1.tif]

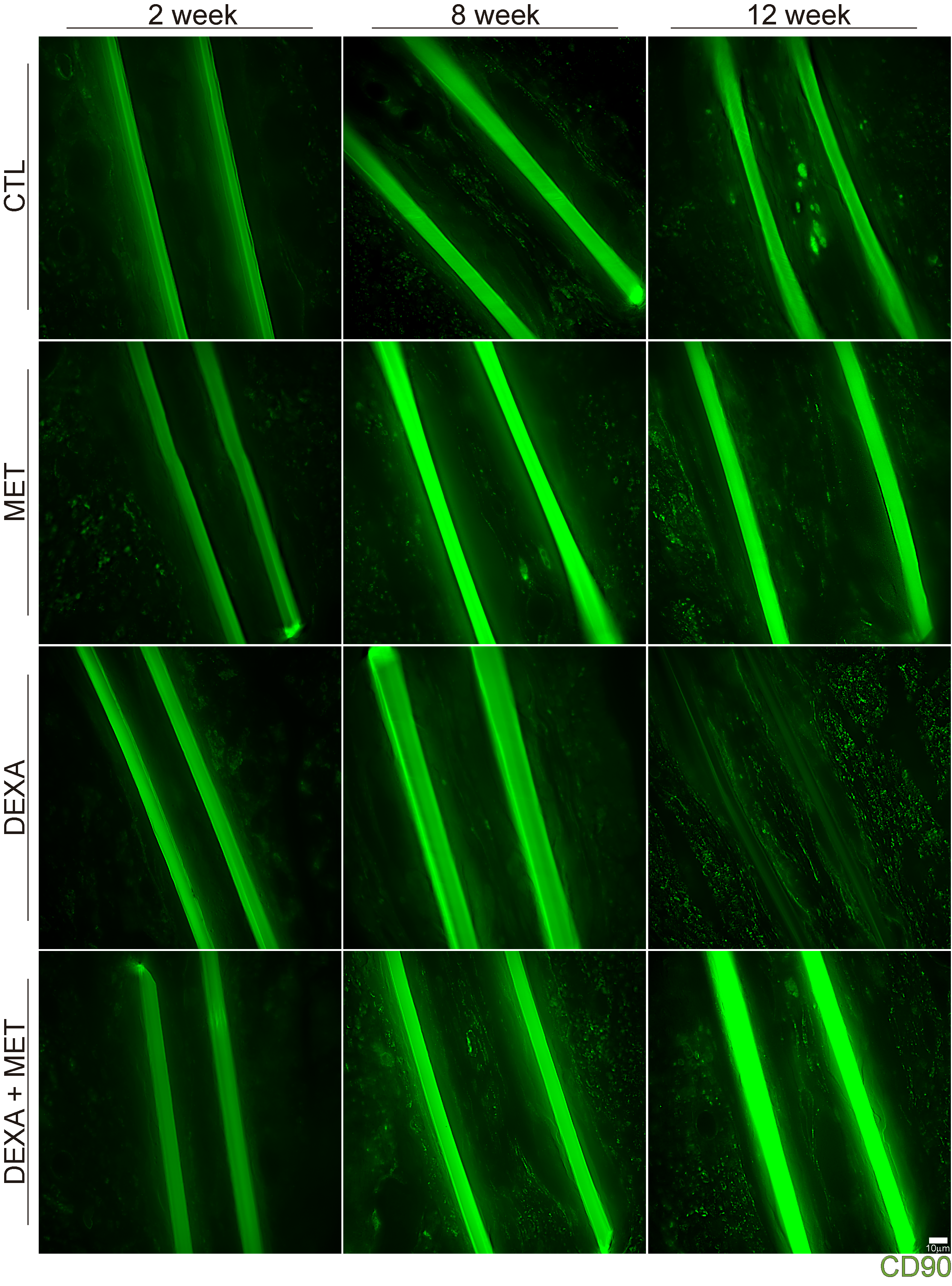

Supplement: Supplementary file 1 [file cells-13-02112-s001.zip › Sup Figure S2.tif]

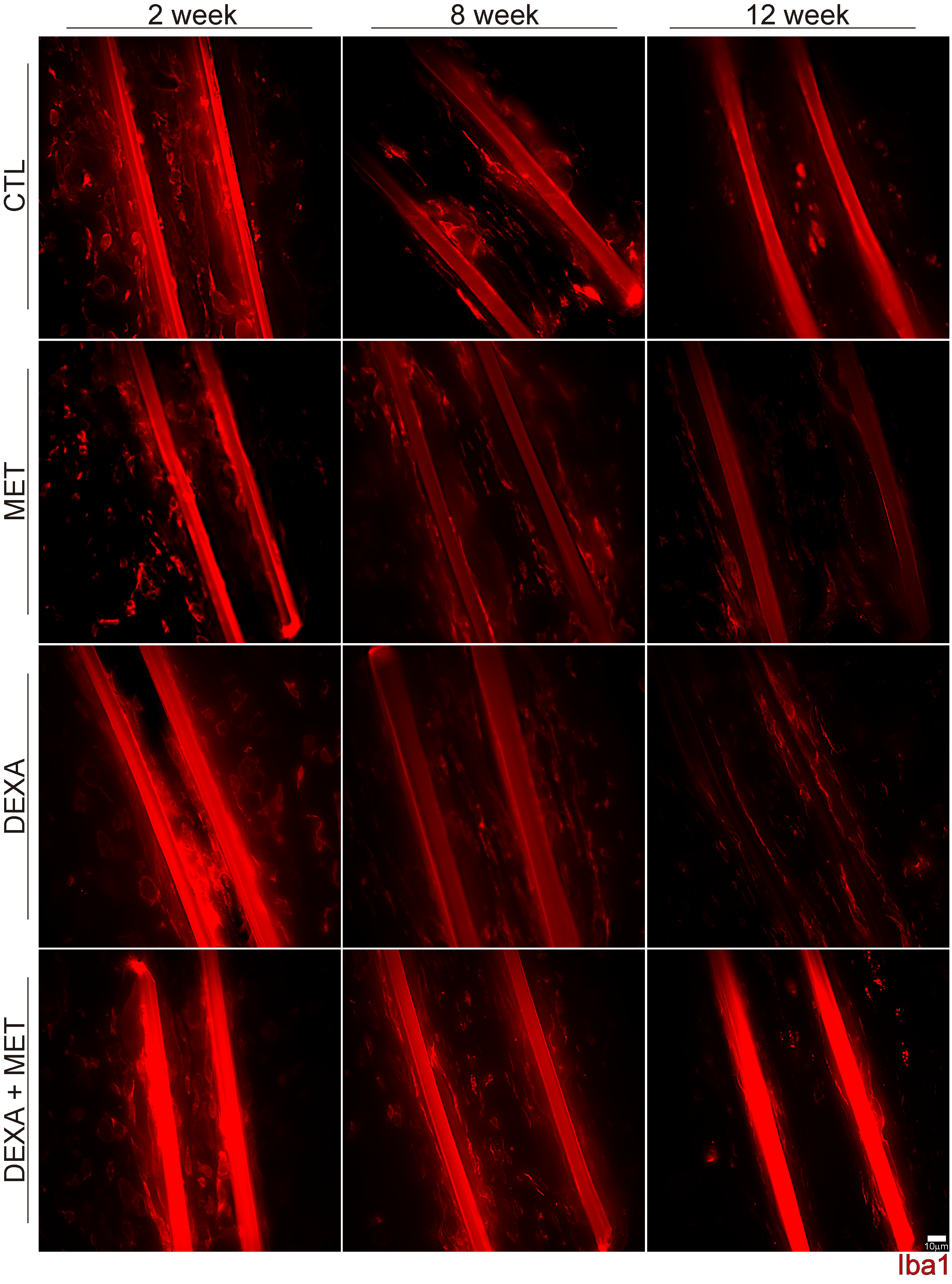

Supplement: Supplementary file 1 [file cells-13-02112-s001.zip › Sup Figure S3.tif]
